# Protective factors for suicidality: a qualitative follow-up of the youth and mental health study cohort

**DOI:** 10.1186/s12889-025-23131-2

**Published:** 2025-05-24

**Authors:** V. Bakken, N. Skokauskas, A. M. Sund, Jannike Kaasbøll

**Affiliations:** Department of Mental Health, Faculty of Medicine and Health Sciences, Norwegian University of Science and Technology (NTNU), Regional Centre for Child and Youth Mental Health and Child Welfare (RKBU Central Norway), Trondheim, Norway

**Keywords:** Suicidal ideations; suicide attempts, Protective factors, Adolescence, Longitudinal study, Qualitative study, Retrospective study, Norway

## Abstract

**Background:**

Understanding suicidality requires a lifespan perspective, yet knowledge of protective factors spanning from adolescence into adulthood remains limited, particularly when examined through qualitative approaches. Our study bridges this gap by exploring retrospective experiences of protective factors for suicidality, as expressed by adults who have faced suicidal ideation (SI) and/or suicide attempts (SA) in their adolescence, and in some cases, later in life.

**Methods:**

Participants were recruited from the Norwegian Youth and Mental Health Study, a representative population-based cohort. Inclusion criteria were prior self-reported SI or SA during adolescence and participation in the latest cohort follow-up (2012). Fourteen individuals evenly distributed by gender aged 38 to 40 participated in this study. Semi-structured interviews were conducted, and reflexive thematic analysis was used.

**Results:**

Four overarching protective factor themes were identified: meaningful relationships and connectedness, support from external sources, positive changes in social-environmental context, acceptance and enhanced understanding. Participants described social and environmental protective factors as particularly influential, shaping individual resilience and adaptive responses, highlighting their significance in suicide prevention efforts across developmental stages.

**Conclusions:**

This study advances the understanding of suicidality from adolescence to adulthood by capturing the lived experiences of those affected. The role of social and environmental protective factors was paramount in shaping adults’ reflections of suicidality and its long-term impacts. It underscores the need for early interventions and further research on protective factors’ evolvement over time.

**Supplementary Information:**

The online version contains supplementary material available at 10.1186/s12889-025-23131-2.

## Background

### Suicidality from adolescence to adulthood

Suicidality—encompassing *suicidal ideation* (SI), defined as thoughts about ending one’s life, and *suicide attempt* (SA), defined as engaging in behaviour with the intent to die—is considered a key risk factor for suicide, the act of taking one’s own life [1]. It remains a persistent global public health concern [2, 3]. In Norway, the prevalence of suicidality and completed suicide is comparable to other high-income countries [2, 4], and national prevention strategies emphasize early intervention and primary prevention among young people [5]. Adolescence is a critical period of onset suicidality [6], however completed suicides progressively increase with age [7]. Adolescence and young adulthood are periods of significant developmental change, including identity formation, emotional regulation and social role transitions, which can increase vulnerability to mental health conditions and suicidality [3, 8–10]. Later in adulthood, socioeconomic, physical -and mental health conditions and family stresses can contribute to suicidality [11]. While there are age-specific risks, suicidality is a dynamic and evolving phenomenon, requiring a lifespan approach to understand how developmental trajectories and external factors influence suicidality over time [12–14]. Adolescent suicidality is often strongly predictive of suicidal outcomes in adulthood [15, 16], highlighting the importance of prevention across all life stages.

Traditionally, knowledge of suicidality has derived from studies focusing on the identification of risk factors among vulnerable or clinical populations, although there are now many studies conducted in community samples [17, 18]. The presentation of suicidality may differ in community populations where identifiable risk factors are less pronounced, as many do not seek mental health care or are reluctant to disclose suicidality due to stigma and other reasons [19–21]. Moreover, suicidality shows a well-documented gender paradox, where females more often report SI and SA, while males have higher rates of completed suicide [22]. Compared to extensive research on risk factors, studies highlighting protective factors for suicidality remain limited [23–25] and are mostly quantitative, cross-sectional studies [17], relying on predefined variables that may not fully encompass their depth and dynamism over time. Therefore, understanding the altering experience of protective factors for suicidality from adolescence to adulthood is important for developing effective prevention strategies.

### Protective factors for suicidality

Protective factors include personal/individual, relational/social and ecological/environmental elements that reduce the likelihood of suicidality [23–25]. This study draws on Bronfenbrenner’s Ecological Systems Theory [26], Positive Psychology [27] and the Developmental Assets Framework [28] to conceptualize protective factors not merely as moderators of risk, but as promotive elements that foster well-being.

Quantitative studies identify self-worth, abilities, and adaptiveness as key protective factors [29–31], consistent with resilience theory, which emphasizes individuals’ abilities to adapt and thrive despite adversity, including reducing suicidality across life stages [24, 32]. Health behaviors, such as physical activity, also play a role [29, 33], though sex differences have been observed [34]. Positive relationships within family, peer, and community settings are frequently identified as protective [29, 32], though their influence varies by age [35–37]. For instance, parental relationships are protective during adolescence [38, 39], while peer [40] and adult social relationships [41] show less consistent effects. Environmental factors, such as school connectedness in adolescence [42] and positive work environments in adulthood [43] can also contribute to reduced suicidality, though these influences remain complex and require further research to clarify their roles across life stages.

Qualitative studies on protective factors for suicidality across life stages are scarce [44], with the only comprehensive review on recovering the desire to live dating back to 2008 [45]. Only twelve qualitative studies met the inclusion criteria in the review for Lakeman and FitzGerald’s review study, and identified studies focused on high-risk or vulnerable samples, including minority groups, inpatients, and bereaved individuals. The synthesis revealed that reconnecting with people and surrounding environments, experiencing “turning points” involving life changing positive events, and developing new coping strategies was pivotal in restoring individuals’ desire to live. More recent qualitative studies have identified additional protective factors for suicidality, including formal and informal social support [46], positive environments characterized by security and acceptance [47], a sense of felt responsibility [48], active resilience development [49] and professional advice [50].

Qualitative studies provide a deep exploration of the lived experiences and complexities of suicidality [51], offering valuable insights to address gaps and uncovering lesser-known phenomena [52]. Qualitative studies may therefore be especially valuable in addressing the gaps in our understanding of protective factors. To our best knowledge, so far, no qualitative study has explicitly investigated protective factors by retrospectively exploring the experiences of suicidality over time among participants from a longitudinal cohort study.

### Objective of this study

The study’s objective is to address existing knowledge gaps by gaining deeper insight into protective factors for suicidality over time, encompassing suicidal ideation (SI) or suicide attempt (SA) as previously reported in adolescence. This is explored with the following question: *What are the retrospective experiences and perceived protective factors related to suicidality*,* as expressed by adults who have faced suicidal ideation (SI) or suicide attempts (SA) in their adolescence?*

## Methods

### Study design

This a qualitative follow-up study (adulthood), from a quantitative longitudinal cohort study (adolescence to young adulthood). Participants were part of the “Youth and Mental Health Study” (YAMHS) [50], a population-based cohort study conducted in central Norway that collected data from 1998 to 2012. Data collection was conducted through four waves using self-administered questionnaires completed by adolescents: T1 (1998, *n* = 2464, mean age 13.7, SD = 0.6), T2 (1999, *n* = 2432, mean age 14.9, SD = 0.6), T3 (2005, clinical subsample, *n* = 345, mean age 20.0), and T4 (2012, *n* = 1266, mean age 27.2, SD = 0.6) [53]. This qualitative follow-up study took place in 2024, when participants were aged 38–40 years old.

SI and SA were measured in the YAMHS questionnaires (adolescence and young adulthood). SI was measured by a mean score from a five-item composite scale, made up by four items from the Moods and Feelings Questionnaire (MFQ) -assessing current and predicted SI among symptoms of depression in adolescents [54, 55], and one item from the Centre for Epidemiological Studies Depression Scale (CES-D) [56] to include more passive SI. The MFQ items were as follows: “I thought that life was not worth living”, “I thought about death or dying”, “I thought my family would be better off without me”, and “I thought about killing myself” and the CED-D item was “I would have killed myself if I had known a way of doing it”. Responses were given on a three-point Likert scale, ‘not true’ (0), ‘sometimes true’ [1] or ‘true’ [2], over the past two weeks. The SI composite scale has shown good reliability in its previous uses in the YAMHS [57, 58]. Mean scores for SI were calculated, ranging from 0 to 2, with scores above 0.25 as the cut-off, indicating elevated levels of SI [55]. SA was measured by a single question deriving from the “Young in Norway” study [59], “Have you ever tried to end your life?” Answers to this item were self-reported using a three-point Likert scale “No, not really” (0), “Yes, once” [1] and “Yes, several times” [2], with only 1 or 2 indicating SA.

### The qualitative follow-up study

#### Interview guide

The semi-structured interview guide was developed based on existing theories [26–28], previous research [29, 32, 33, 42], as well as input from advisors with lived experiences. The guide included both primary and prompt questions to guide the interview process (Additional file 1). Suicidality context was explored through questions such as: “What was your experience of SI/SA in adolescence? What was going on in your life at the time? Have you also experienced SI/SA later in life?”. This followed by a focus on protective factors at individual, social and environmental levels, for example: “What was the most important thing that helped you when experiencing SI/SA in adolescence (and later in life if applicable)?”, “Can you describe whether any people or other things around you that helped?”, “Can you describe if there was anything that you did that helped?”. Participants were encouraged to reflect on the protective factors they found most important during adolescence, but also at other timepoints and, if applicable, at later life stages. To conclude, participants were asked to share insights on potential supports that might assist future adolescents facing similar challenges.

#### Participant selection - inclusion criteria

To be eligible for the study, participants needed to have reported elevated suicidal ideation (SI scale score above 0.25) and/or past suicide attempt attempt (score of 1–2 on the SA question), as adolescents (at T2, 1999) [55]. In addition, participants were required to have taken part in the study as young adults (at T4, 2012) to access necessary contact information to identify participants, including personal ID numbers and previous addresses. Participants were only included in the study if they as adults, at the age of 38–40, were able to recall experience of adolescent SI or SA. This decision was made based on ethical considerations, where it could cause unnecessary harm if participants were fully informed of their previous responses involving suicidality. A total of 207 participants were identified as eligible for the present qualitative study (147 women and 60 men). Upon request, the Norwegian Population Registry provided verified and updated contact information (i.e., updated names, addresses, other statuses) for these participants. Three individuals had died since T4, and addresses could not be verified for 14 participants due to missing or incomplete personal ID-numbers, or unregistered Norwegian addresses. This resulted in 190 eligible and verified participants.

#### Recruitment process

**Fig. 1:**
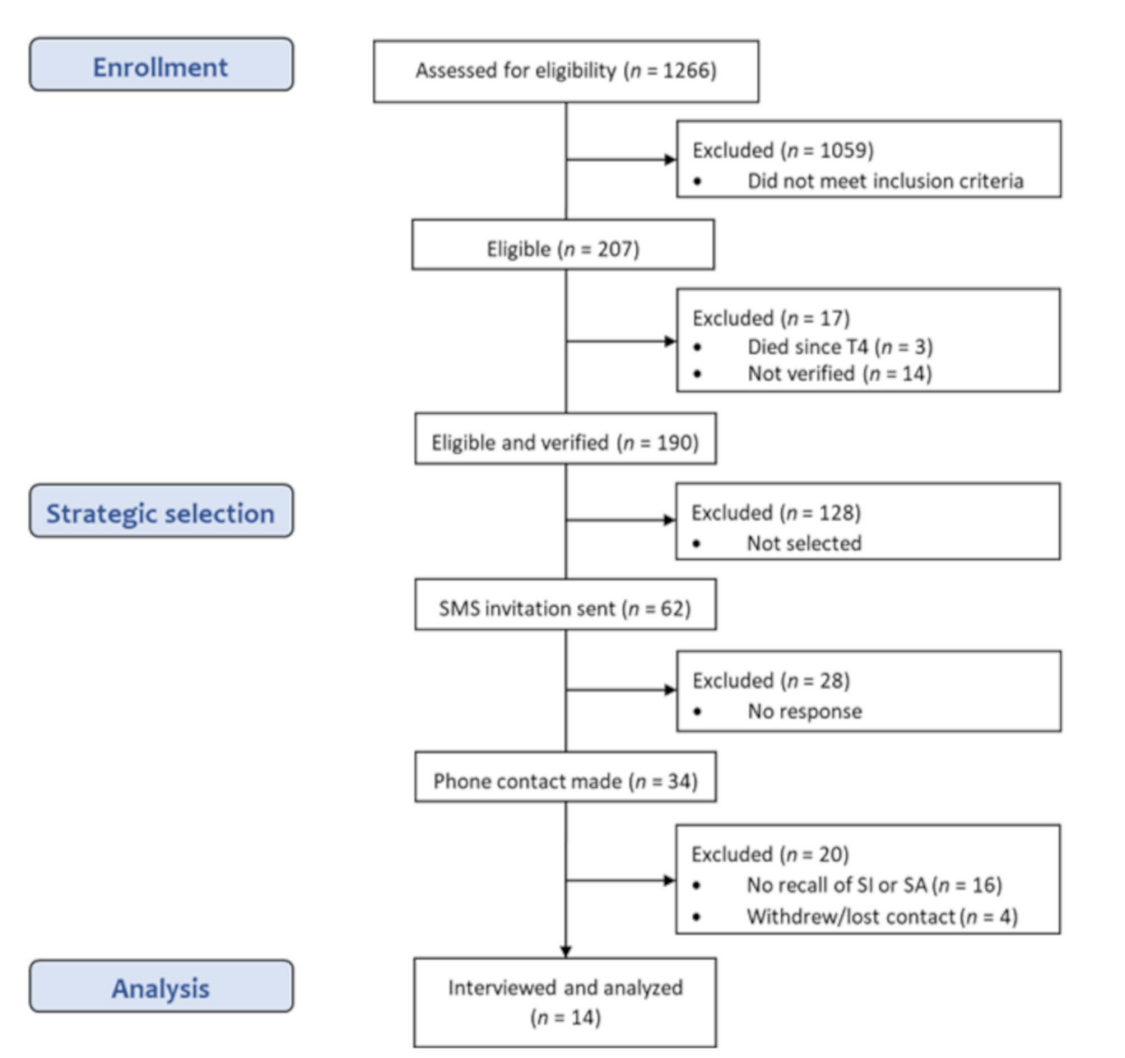
Flow chart of participants, SI = suicidal ideation, SA = suicide attempt

Recruitment commenced in January 2024 and involved strategic selection, which is purpose-driven to ensure diversity in specific characteristics relevant to the research question. In this study this meant obtaining variation in gender, location (urban/rural areas), and type of suicidality (SA/SI). Each week eight to ten eligible participants received an invitation to take part via SMS. Group-based recruitment was conducted to avoid rejecting potential participants if the target group size was exceeded. Those who responded to the SMS were contacted promptly, and those who did not received a follow up phone-call a week later. During the initial phone-calls, participants were further informed about the study and screened to determine whether they could recall SA/SI during adolescence. They were not asked to recall questionnaire responses, but instead their experiences, typically framed as: “We are seeking former YAMHS participants who can recall experience of suicidality during adolescence, including thoughts and/or attempts. Have you had such experiences?”. Only those who could recall adolescent experiences of suicidality, and provided their consent were finally included as participants in the study. Figure 1 provides a flow chart of the recruitment process.

*Flow chart of participants*, SI = suicidal ideation, SA = suicide attempt.

Through repeated recruitment in groups of 8–10 participants at a time, a total of 62 potential participants received the SMS invitation. Phone contact was successfully established with 34 of them, while 28 did not respond. There were 16 individuals contacted by phone who did not identify with having had experience of suicidality in adolescence. Four individuals could recall experiences and were initially interested in participating, but did not end up taking part in the study; One withdrew due to time constraints, and three did not attend the scheduled interview -and contact was lost. Interviews were conducted until adequate saturation was accomplished and added information stopped emerging. This resulted in interviews with a total of 14 participants, seven women and seven men, by the end of March 2024.

#### Data collection

Participants who during the phone call met inclusion criteria and consented to take part in the study were given the option of being interviewed online, by telephone, or face-to-face. Eight interviews were done online (Microsoft Teams) and six by telephone. The duration of interviews was set at approximately one hour, and most overheld this timeframe. An Olympus Digital Voice Recorder (VN-711PC) was used to record interviews, which were transferred to an encrypted data file promptly after the interview. Interviews were transcribed verbatim with aid from an offline version of the program Whisper, to ensure data privacy and prevent exposure of sensitive information to external servers. This process also involved removing any person-identifiable information contained within transcripts (i.e., anonymizing names and locations). Exemplary quotes were translated by the first author and verified by co-authors.

#### Data analysis

Reflexive thematic analysis (RTA) was utilized to interpret the qualitative data in this study. RTA enables in-depth exploration of accounts of subjective experiences, where the aim is to seek meaning through the generation of “themes” [60], which are broader patterns of ideas and insights that capture meaning behind data in relation to the research question. Analysis in the present study has distinguished between “overarching themes” as the main and most prominent patterns, and “subthemes” which are specific patterns that are integral to the overarching themes. Themes were generated from “codes” which could be described as labelled or tagged segments of data which capture pieces of information. Reflexivity is central to RTA, meaning that the first author (VB) has thoroughly interrogated her active role in the interpretation and production of knowledge throughout the research process [61]. Keeping a reflexive diary has been continuously utilized for this purpose. Some initial analyses were done during data collection, however, most analysis was conducted after all data had been collected. Qualitative data was managed and coded using NVivo software -version 14, Microsoft Word, and mind-maps.

The first author (VB), a PhD-candidate at Department of Mental Health, NTNU, had main responsibility for the analysis, which passed through the six phases of RTA as described by Braun and Clarke [62], conducted with flexibility in sequence. VB has previous experience conducting qualitative research and thematic analysis, as well as clinical work with individuals affected by suicidality. Her gender and regional dialect were naturally apparent to participants, though no additional personal characteristics were disclosed. Her role in the project was clearly communicated. Debriefs and discussions with co-author (JK) took place at each phase of analysis, including after each interview, whereby VB and JK talked about how the interview went, main topics and any difficulties. *Phase 1*, involving data familiarization, started during the conduction of interviews combined with hand-written notes. All Whisper-output transcriptions were quality-checked and compared with audio recordings. During manual amendments, further familiarity notes were taken. In *Phase 2* data was systematically coded using NVivo, through labelling/tagging phrases and sentences that captured specific features of information. An inductive approach was used, meaning that codes were generated from the data itself, and not pre-determined topics or frameworks. However, some codes were deductive and based on research question topics covered in interviews, i.e. social factors. Generated codes were mostly latened (interpretive and beyond what is explicitly stated), however, a few were semantic (directly captured from words/phrases in transcripts). *Phase 3* was an active process whereby several codes were grouped together, based on relevance and connections to each other. These grouped codes were used to generate initial themes that captured meaning relating to the research question. Visualization tools were utilized for this purpose, for example inspecting code frequencies and exploring connections between codes. *Phase 4* required going back to the full dataset collectively. From considering grouped codes, themes (overarching -and subthemes) were developed and reviewed. Individual participants’ codes and themes were checked against the entire dataset, exploring nuances. *Phase 5* focused on refining, defining, and naming themes. This involved detailed and in-depth interrogation of all past phases and coherent analytic developments. Ideas deriving from discussions with co-authors were also revisited and critically evaluated. The purpose of the final phase was determining the core focus of each overarching theme and accompanying sub-themes. Finally, in *Phase 6* analysis and extracts were contextualized considering existing research through article-reporting.

The study adheres to the COREQ (Consolidated Criteria for Reporting Qualitative Research) 32-item checklist [63], ensuring transparency and rigor in the reporting of qualitative research. A completed checklist is provided in Additional file 2.

#### Consultation and involvement of advisors with lived experience

External advisors with lived experience of suicidality played an integral role in the qualitative follow-up study. They were actively involved in the project from its inception, contributing to the funding application and subsequent stages of the research process. Two representatives of “Landsforeningen for Selvskading og Selvmord” (LFSS; English translation: The National Association for Self-Harm and Suicide) contributed to developing and refining the interview guide and informed consent form. Additionally, a third independent advisor with lived experience of suicidality (not affiliated with LFSS) participated in a pilot interview, and provided valuable feedback on the recruitment process, the overall interview experience, and the structure of the interview guide.

### Ethical considerations

All previous waves of the YAMHS have received ethical approval from the Regional Committee for Medical and Health Research Ethics (latest approval, REC: 2011/1454). The present qualitative follow-up study has been approved by REC (ID: 424436). Due to the sensitive nature of the research topic, thoroughly thought-out safety measures were introduced to minimize risks and ensure wellbeing of all participants. This included screening by the phone which enabled identification of current active suicidality, but also whether participants were comfortable sharing their experiences. In the case of encountering someone with active or chronic suicidality at any stage of the project, available support (helplines and emergency numbers) details were specified in the informed consent form, and directly to participants by telephone if needed. All participants provided informed consent and were compensated with a gift-card valued 1000 NOK (Є86, $90) for their contribution and time.

## Results

### Participant characteristics

Information about participants’ gender, age, family situation, and reported SI/SA at T2 (adolescence) and at the qualitative follow-up study (adulthood) are presented in Table 1. The sample consisted of 14 adult participants, all of whom had experienced suicidality during adolescence. Half of the participants identified as female. At the time of the interviews, participants were between 38 and 40 years old. Seven participants were married or in a relationship, while the remaining seven were either separated or single. Eight participants had children. All participants acknowledged experiencing SI during adolescence. However, the recall of SA was less consistent with previous self-reports; some confirmed SA, others described non-explicit events that could imply SA, and a few denied having any SA experiences or stated that it occurred only after adolescence. No participants described more than one SA from adolescence to adulthood. Although participants were primarily interviewed about adolescent suicidality, eleven also affirmed later experiences following adolescence (i.e. early adulthood, adulthood). Three of these had chronic SI from adolescence into adulthood; however, two noted significant improvement in managing these thoughts after identifying the appropriate antidepressant medication. In the qualitative follow-up, bullying, exclusion, and social isolation were the most frequently cited contextual factors contributing to suicidality during adolescence. Some participants also identified family difficulties, relationship dissolution, substance use, and bereavement as contributing factors. However, to maintain participant anonymity and confidentiality, these experiences are not included in the table.

**Table 1:** Participant characteristics

Characteristic		Number
**Gender**		
Female		7
Male		7
***Age (years)***		
38		5
39		5
40		4
***Family situation***		
Single		4
Partner		2
Single/separated, with children		3
Partner/married, with children		5
***SI or/and SA reported at T2***,*** adolescence (14.9 mean age)***		
SI		4
SA, once		3
SI and SA, once		3
SI and SA, more than once		4
***SI or/and SA recalled at follow-up***,*** adulthood (38–40 years)***		
SI		7
SI and SA		3
SI and (SA)		4

SI = Suicidal ideation, SA = Suicide attempt, (SA) = Suicide attempt not explicitly recalled, but participant described an event that could indicate potential past SA

### Overarching themes

Four overarching themes representing protective factors for suicidality among participants were identified (1) meaningful relationships and connectedness (2) support and help from external sources (3) changes in the social-environmental context, and (4) acceptance and enhanced understanding. While the primary focus of this study was protective factors during adolescence, relevant insights from later life stages were also included. Within these themes, distinct sub-themes were identified, highlighting the diverse ways these protective factors operated in participants’ lives. Together, these themes provide a nuanced understanding of the personal, relational, and environmental influences that helped participants navigate periods of suicidality. The overarching themes and associated sub-themes are outlined below. An overview of all themes and additional illustrative quotes from interviews are available in Additional file 3.

#### Meaningful relationships and connectedness

Most participants expressed experiencing the importance of supportive family and friend relationships, which provided emotional security and served as protective factors for suicidality. Within this theme, three sub-themes were identified: family support and emotional connection, close friendships, and not wanting to hurt loved ones.

##### Family support and emotional connection

Many participants described close relationships with family members, especially parents and siblings, as a critical protective factor. These bonds offered emotional comfort and a sense of security, where participants professed that family were experienced as unconditionally supportive, available and present in their lives. While a few participants felt comfortable seeking emotional support (e.g. disclosing SI or SA directly), others preferred to seek practical support from family members (e.g., asking parents to communicate with teachers about school issues). Participants expressed that emotional support nurtured their resilience, whereas practical support alleviated external challenges. Some participants expressed that siblings were more approachable than parents, and a few also turned to extended family (e.g. cousins) for support. For those who experienced suicidality later in life, positive romantic relationships (partner, spouse) were important in alleviating suicidality. To illustrate the importance of family connections, one participant reflected on support received from her mother following disclosure of difficulties that had contributed to SI:

*It was just about having shared it with someone*,* so she knew (…) Because if at least one person or two wants you in the room*,* wishes for you to be here and say hi to you and looks after you– then you can get through it. (Emma*,* experienced SI during early-mid adolescence)*

##### Close friendships

Many participants shared that close friendships offered significant protection for suicidality during adolescence. Typically, this protection came from meaningful connections with one or a few long-term close friends - individuals who provided deep understanding, comfort and support. Like family relationships, participants described variations in what “connectedness” meant to them. For some, having friends with whom they could openly share their emotions and thoughts with was essential. For others, the primary benefit lay in the companionship and positive distractions that came from simply spending time with friends. These connections, whether emotional or activity-based, played a vital role in fostering a sense of belonging and relief from distressing thoughts. The significance of friendships is illustrated by one participants’ description of his two closest long-term friends:

*For me it meant everything. It is something I think about daily. I haven’t actually…not yet thanked them for it. I think they know. But…it has meant everything to me. They included me*,* came to my home*,* and picked me up. They brought me out to do things. Got my thoughts on something else. (David*,* experienced SI from mid-adolescence to young adulthood and one SA in late adolescence)*

##### Not wanting to hurt loved ones

Meaningful relationships with family and friends were strongly linked to a reluctance to cause harm to loved ones. Although some participants acknowledged that more openness with loved ones could have alleviated suicidality, most highlighted that avoiding concern or harm to others was more protective. Alongside an unprecedented sense of connectedness, exposure to suicidality, such as through friends’ suicides, was explained as reinforcing of this protective factor for several participants. The awareness that their suicidality would have a profound impact on loved ones deterred them from further engaging in SI or SA during adolescence and continued to serve as a protective factor into adulthood. In adulthood, several participants with children described that fear of inflicting emotional pain on them prevented engaging with thoughts of suicide. Not wanting to hurt others is illustrated by one participants’ contemplation:

*Mainly I think about how my mother would have reacted. It would just be hurtful. I’ve known many friends who took their lives*,* and their mothers*,* they have been completely devastated. It is horrible. (Thomas*,* experienced SI chronically from adolescence through to adulthood)*

#### Support and help from external sources

The second theme highlights the role of external help from individuals outside the participants’ immediate social circles, providing non-judgmental and unbiased support when facing suicidality. Two sub-themes were identified within this theme: external helpers; and seeking professional help.

##### External helpers

Several participants described active help-seeking as particularly challenging during adolescence. However, those participants who had reached out to parents for help earlier often described receiving more consistent external support throughout their adolescent years. Participants often declared appreciation for the support from external, “more neutral” individuals such as acquaintances, teachers, school nurses, and coaches. These external helpers were not part of participants’ closest personal networks but took notice and offered non-judgmental support when needed. Participants expressed that this outside support helped them feel seen and heard, regardless of not having actively sought help. A few participants were prevented from a potential SA by external helpers. This was described by one participant, who was approached by an acquaintance when standing on a bridge and contemplating suicide:

*He started talking to me. I think he saved me from doing something stupid that evening. I was certain that my life was going to end there and then. (Leah*,* experienced SI during adolescence and recalled a potential SA at this time)*

##### Seeking professional help

Several participants had experience seeking professional psychological support, though many did not pursue this until late adolescence or adulthood. Some recalled negative experiences when initially seeking help, describing feelings of not being taken seriously or supported, which they found embarrassing. This often led to delays in seeking professional support again. These delays were also attributed to perceived stigma, and limited knowledge about mental health and available services. Engaging with mental health professionals, such as psychologists or psychiatrists, was described by participants as enabling them to process difficult past experiences, gain practical self-help strategies, and receive unbiased support and guidance. For some, these interactions also led to identification of previously undiagnosed mental health conditions, such as anxiety or depression, which brought new understanding and direction for their well-being. While most participants who received therapy found it to be highly beneficial, a few noted that medication also had a substantial positive impact on managing symptoms. When thoughts of suicide resurfaced in adulthood, one participant reflected on the professional help he had received:

*It was speaking to the psychologist and getting the third-party view. And it was also good to have a GP during that time*,* who also was a third-party who listened well and took me seriously. (Benjamin*,* experienced SI in early-mid adolescence and had later experience of SI in adulthood)*

#### Changes in the social-environmental context

This theme captures how positive shifts in social environments, including changes in surroundings or participation in shared activities, provided participants with a sense of belonging and identity outside of their personal challenges. Two sub-themes were identified: Engagement in activities and sense of belonging; and A new start.

##### Engagement in activities and sense of belonging

Having places to belong and connect with others outside of everyday settings of home or school/work, was a central protective factor. Participants shared that engaging in activities, such as sports, extracurricular programs, travel, or pursuing hobbies and special interests, positively impacted their mental health, reduced suicidality and enhanced overall well-being. These activities provided a sense of inclusion and allowed participants to feel part of something larger, fostering belonging and connection with others who shared similar interests. For some, involvement in these activities shifted their focus, offering a welcome distraction from negative thoughts and feelings. Additionally, many participants noted that certain activities brought physical benefits, leading to greater relaxation physically and psychologically. Although some organized activities became less frequent with age, engaging in physical activities or hobbies, were also important protective factors for suicidality in adulthood. Participants expressed that engaging in these pursuits often led to a sense of mastery, which in turn appeared to improve self-esteem, self-worth, and social competence. One participant captured this sentiment well, stating:

*I think*,* it was the unity in relation to football and that stuff. At least you felt that you had a sanctuary where you mastered something. And at the same time that you had a social community and feeling that you could meet people and hang out in your free time. That you had someone to share a special interest with. And they used a lot of their time and dedicated their time in a similar way to you (Hugo*,* experienced SI during adolescence*,* which returned in his twenties -when he also had a SA)*.

##### A new start

For many participants, engaging in new activities was often marked a fresh start in a new context. Some participants described that life alterations, such as changes to the family structure or relocating, could be experienced as challenging and uncertain to begin with. However, a change was by most characterized by stronger sense of identity and adaptability. Starting a new school, in particular, was described as a transformative turning point in adolescence, especially for those who had previously been bullied or socially excluded. This transition enabled participants to leave behind a negative social environment and opened opportunities for positive change. The new start frequently brought improved school support and introduced participants to more like-minded and mature peers, often through shared activities. In cases where suicidality resurfaced in adulthood, initiating a new start was often described as protective. This included actions such as travelling or making active life changes trough relocation, switching fields of study or changing jobs. Participants described these changes as fostering a sense of renewal, healthier social connections, and an improved sense of belonging. As one participant reflected following a new start, involving a change of school:

*Things became much better*,* I got friends. The environment was larger*,* offering more space to be ourselves. (…) I think it was that I felt I had belonging. That I actually belonged somewhere. And that I fitted in. To have mutual interests*,* and community. (Alice*,* first experienced SI and SA in late childhood*,* and some continued SI in adolescence)*

#### Acceptance and enhanced understanding

The fourth theme underscores how participants’ acceptance of their past experiences, along with their reflections on these, led to enhanced understanding of themselves and the world around them. This process was described by participants as rebuilding themselves and contributing to the development of resilience and a more positive self-concept. Two sub-themes were identified here: Reflective growth through adversity– resilience; and Enhanced understanding and societal awareness.

##### Reflective growth through adversity– resilience

Many participants retrospectively described their adolescent years as a profoundly challenging period. However, they noted that enduring these hardships fostered resilience, adaptability, and an improved sense of self. For most, facing adverse experiences gradually helped build their ability to cope and adapt, ultimately leading to a strengthened self-perception. Participants had processed these experiences over time from adolescence to adulthood, and expressed that acknowledging and accepting their past struggles was essential in helping them recognize their own resilience and personal growth. As one participant shared:

*It’s the whole process of not giving up. You must fight. Most of the things you do is difficult. So*,* it’s something about the sense of mastery*,* to have struggled with something for a long time. That can easily be transferred to other aspects of life. To have spent thousands of hours trying and learning something*,* and succeeding in the end. So that is a very valuable lesson to have later in life. (Henrik*,* experienced SI chronically from adolescence through to adulthood)*

##### Enhanced understanding and societal awareness

Over time, several participants described developing a deeper understanding of their adolescent struggles, particularly regarding suicidality. Hindsight and life experience allowed them to contextualize their feelings within broader negative influences and circumstances from that time. Many described how increased personal and societal awareness about mental health and suicidality had been protective factors, helping them recognize that these challenges were complex and multifaceted. They expressed that the evolving openness and acceptance surrounding mental health had been instrumental in reshaping their attitudes. Instead of thinking about themselves as someone “abnormal” or as “a black sheep” for experiencing difficulties and suicidality, they became aware that they were not alone. Increasing understanding and awareness as adults also provided participants with valuable reflections of what might have been helpful during their adolescence. Many described that understanding adolescence as an inherently challenging period across several aspects of life has been important. As adults, having gained awareness of supportive resources and learning to maintain a positive future outlook were critical factors that participants alleged as contributors to suicide prevention among today’s adolescents. One participant reflected when considering his own experiences in relation to future adolescents:

*You’re in a phase of life where there’s an awful lot of hormones*,* there are many feelings. You are uncertain*,* and others are uncertain too. Life is pretty long*,* and this phase you’re in now is a pretty short one. Try to help identifying thoughts about what life can hold afterwards. Everything is much greater. (Nicolas*,* experienced SI from late childhood to late adolescence and recalled potential SA during adolescence. Has also experienced SI in adulthood)*

## Discussion

This study examined protective factors for suicidality as recalled by adults reflecting on their adolescence, while also considering relevant experiences later in life. The findings provide valuable new insights into how these factors supported participants during a critical developmental period. No previous qualitative research has retrospectively explored protective factors for suicidality over time within the context of a longitudinal cohort study, making this investigation uniquely significant. Participants emphasized the significance of social and environmental protective factors, which emerged as a key finding in this study. Family and friendships were emphasized as vital sources of support that reduced suicidality by contributing participants’ sense of belonging, resilience and mental well-being. Notably, some found external helpers more approachable, showing that connections beyond one’s inner circle also can serve as a valuable protective influence during times of suicidality. Individual protective factors, such as positive self-perceptions and adaptability were frequently described by participants as being deeply influenced by the social interactions and environmental contexts experienced throughout adolescence and into adulthood. This perspective underscores the importance of considering both social-environmental contexts and developmental shifts from adolescence to adulthood when assessing protective factors for suicidality across the lifespan.

Findings align with existing research highlighting the protective role of meaningful relationships, commitment and felt responsibilities in reducing suicidality [29, 32, 48]. However, our study’s retrospective design revealed additional nuances, such as participants’ concerns about the potential impact on loved ones (including parents, siblings, partners and own children), which both supported their will to live, yet often deterred them from disclosing their struggles. Similar complexities have only been explored qualitatively [48], and warrant further investigation. Consistent with prior studies, individual and more internalized protective factors like self-perception -and efficacy, resilience and adaptability were essential aspects of reducing suicidality during transitions from adolescence into adulthood [24, 29–33]. However, in contrast to research depicting individual assets as fixed protective factors, participants in this study emphasized that these primarily evolved alongside changing social environments, shifts in societal perceptions, and through meaningful relationships. Changes in external environments, such as transitioning to a more positive school setting, were especially critical in providing opportunities to cultivate individual coping strategies (i.e. positive reframing) and discover new perspectives on self-worth.

Cognitive maturation and life experience also appeared influential, as participants described how earlier dysfunctional thinking patterns, such as catastrophizing or black-and-white thinking (“life is hopeless,” “I’m in a black hole”), were gradually challenged by experiences that disproved these negative thoughts and provided hope. The dynamic interaction between individual and external protective factors reflects Joiner’s Interpersonal Theory of Suicide [64], which underscores the importance of social connectedness in addressing and reducing perceived burdensomeness and thwarted belongingness in suicidality. While positive life “turning points” have been identified in qualitative research [45], they remain underexplored in quantitative suicide studies.

Suicidality is often conceptualized in modern society as an issue only affecting vulnerable people, where targeting individual risk factors is the preeminent to prevent suicidality [65]. This study’s findings suggest that fostering protective factors is possibly essential to efficient prevention at a population level, and that it requires consideration of both internal and external resources, with positive social relationships and environments serving as a scaffold for individual growth and well-being. This distinction from previous notions can also be understood in the context of past studies predominantly utilizing cross-sectional designs, limiting insight into self-reported protective mechanisms and their dynamics over time. In this qualitative study participants reflected on their experiences from a more mature perspective, revealing the diverse ways in which protective factors contributed to reducing suicidality over time. This retrospective viewpoint complicates direct comparisons with earlier research and highlights the evolution of protective factors throughout development. The emphasis on social and environmental factors may stem from memory processes, especially the availability heuristic– meaning that concrete social events are easier to recall, compared to abstract internal states [66]. As individuals age, they often construct narrative identities that prioritize external influences while minimizing less visible internal factors [67]. As such, recognizing the interconnectedness of individual, social, and environmental protective factors is crucial in understanding their complex roles in suicidality.

### Implications for practice

School-based programs like Youth Aware of Mental Health (YAM) [68, 69], have been effective in addressing stigma and reducing suicidal outcomes. Incorporating protective factors into such interventions could enhance school-class and peer connectedness and acceptance, as well as offer long-term benefits [31]. While some participants noted positive impacts from environmental changes, such as relocating (i.e. new school/workplace) schools, these options are not always feasible and may introduce additional stress. Therefore, prioritization and strengthening of accessible, confidential support systems, such as helplines/chat and counselling services, suitable for early to middle adolescents, can provide vital assistance to individuals reluctant to reach out to family or friends, thereby helping to reduce suicidality both at present and in the future.

The complex interplay of protective factors for suicidality highlights the importance of fostering supportive relationships and strengthening social bonds within families, friendships, and beyond. Participants emphasized that fears of burdening loved ones could deter suicidal actions, yet these same relational dynamics sometimes hindered open discussions due to stigma and concerns about negative repercussions. This underscores the need for stigma reduction and promoting of open family conversations about mental health [70]. As expressed by participants, improved societal understanding and awareness, had over time protected them from suicidality by challenging stigma driven negative self-perceptions.

### Strengths and limitations

Strengths and limitations of this study are intertwined. Being a cohort retrospective study, it offers unique advantages in reviewing individual exposures and outcomes [71]. This type of follow-up extension design is especially valuable for supplementing intriguing quantitative findings with in-depth qualitative exploration [72], in this study focusing on protective factors. Unlike cross-sectional studies, this retrospective, reflective approach enabled exploration of protective factors that persisted or evolved over time. Although all participants experienced suicidality in adolescence, many also faced SI or SA later in life. Similar protective factors were relevant into adulthood, though their expression and impact varied. Future research should employ large-scale, longitudinal, mixed-methods studies to examine how protective factors evolve, adapt and interact across age-groups and developmental stages, providing a more nuanced understanding of their influence over time. Considering the well-known gender paradox in suicidality, additional exploration of potential gender-specific protective factors would also be highly valuable to strengthen future prevention. Although transferability is limited by its sample from participants of similar backgrounds (i.e. ethnicity, culture), the insights gained are valuable for suicide prevention efforts and future research in the field. Recall bias is a consideration, as some participants did not affirm previous adolescent SA, though all acknowledged experiences of SI. This may reflect adaptive and positive memory processes [73] and the influence of retrospective recall biases [74, 75]. Finally, the qualitative analysis relied primarily on the first authors’ interpretation, though this was subject to supervisory oversight to ensure rigor in analytic processes.

## Conclusions

This study underscores the importance of social and environmental protective factors in shaping adults’ reflections on adolescent and later suicidality. Moreover, it highlights the long-term impact of early-life suicidality and emphasizes the need for early, universal intervention strategies to promote resilience and reduce stigma. Findings are also relevant for prevention strategies at secondary and tertiary level, offering clinical care to those with recurring or chronic suicidality. These findings call for further mixed methods research exploring how protective factors evolve across developmental stages to inform effective prevention strategies.

## Electronic supplementary material

Below is the link to the electronic supplementary material.

Supplementary Material 1

Supplementary Material 2

Supplementary Material 3

## Data Availability

The datasets used and analyzed in the present study (interview transcripts) are not publicly available to protect the anonymity of participants. However, data may be made available upon reasonable request from the project leader and corresponding author, J.K. at the Norwegian University of Science and Technology (NTNU), Department of Mental Health, Regional Centre for Child and Youth Mental Health and Child Welfare.

## Abbreviations

SI: Suicidal ideation(s)
SA: Suicide attempt(s)
YAMHS: Youth and Mental Health Study
