# Supplementary material for: Protective factors for suicidality: a qualitative follow-up of the youth and mental health study cohort
Source: BMC Public Health. 2025 May 24;25:1920. doi: 10.1186/s12889-025-23131-2 (PMC12102906; doi:10.1186/s12889-025-23131-2)
Supplement: Supplementary file 1 — Supplementary Material 1 [file 12889_2025_23131_MOESM1_ESM.docx]

**Additional file 1**

**Interview guide**

**«From adolescence to adulthood – protective factors for suicidality»**

**Interview information:**

| **Date / time:** |  |
| --- | --- |
| **Location:** |  |
| **Interview duration:** |  |

**Participant information:**

| **Pseudonym:** |  |
| --- | --- |
| **Gender:** |  |
| **Age:** |  |
| **Marital status (children):** |  |

**SEMI-STRUCTURED INTERVIEW GUIDE:**

| **Primary questions:** | **Prompt questions:** | **Notes:** |
| --- | --- | --- |
| Repeat important information from the informed consent form (any questions about this?).  Complete participant information (above). |  |  |
| As an adolescent you took part in the “Youth and mental health study” (YAMHS), which involved completing a questionnaire at school.  (*show copy of the YAMHS questionnaire from 1999*)  Can you tell me a bit about how your adolescence was for you? | What did life look like back then?  Can you tell me a bit about how things were at school?  Can you tell me a bit about how things were with friends?  Can you tell me a bit about how things were at home and in your family?  Can you tell me a bit about hobbies you had or activities you enjoyed?  What did you enjoy about adolescence?  What was not enjoyable about adolescence?  Any particular experiences or life lessons from adolescence that you brought with you into your adult life? |  |
| We are interviewing those who can remember having experienced suicidal thoughts or/and suicidal behavior as an adolescent.  What can you remember about your suicidal thoughts during adolescence?  Did you experience suicidal behaviors during adolescent (harmed yourself with intention/wish to die, a suicide attempt?)  Have you experienced suicidal thoughts or/and behaviors after adolescence, later in life? | Suicidal thoughts:  How often? How long in duration?  Can you remember when/where you had these thoughts?  Can you tell me a bit about the contents of these thoughts?  Did you have thoughts involving concrete plans about ending your life?  Behavior:  What happened?  Did it happen more than once?  Can you tell me when and where it happened?  What did you think about what had happened afterwards?  (if yes, same prompts as above) |  |
| What was it that stopped the thoughts/behavior that time? (and, later in life *if it also happened then*)  What was that helped or protected you?  (*Note; If both thoughts/behavior, ask prompts separately)* | Was there anything you consciously/with awareness did yourself, -what was it?  Was there something you unconsciously/without awareness did yourself, that you in hindsight think stopped it, -what?  Did someone else consciously/with awareness do anything to stop you when it happened, who + what did they do?  Did someone else unconsciously/without awareness do anything that you in hindsight think stopped it, who + what did they do?  Was there anything happening around you that you think might have helped or protected you? |  |
| What are your reflections about the suicidal thoughts/behavior in hindsight – later in life (young adult/adult)?  What would you have told yourself – if you could go back in time and meet yourself as an adolescent, when you had these thoughts/behavior? | What do you make of it as an adult?  What thoughts and feelings have you had about it? |  |
| *Final questions:*  What would you have said, if you had encountered an adolescent at present who experienced suicidal thoughts/behavior?  Summary – What are the most important things that you think has helped or protected you from suicidal thoughts/behaviors (adolescence, *and later*)? What would you highlight?  Anything else you wish to add or say?  Do you have any questions for me before we finish?  *I wish to thank you for participating in this interview study. Please do not hesitate to contact me or Jannike Kaasbøll if you have any questions in the time after the interview.* |  |  |
