# Supplementary material for: Protective factors for suicidality: a qualitative follow-up of the youth and mental health study cohort
Source: BMC Public Health. 2025 May 24;25:1920. doi: 10.1186/s12889-025-23131-2 (PMC12102906; doi:10.1186/s12889-025-23131-2)
Supplement: Supplementary file 2 — Supplementary Material 2 [file 12889_2025_23131_MOESM2_ESM.docx]

# Additional file 2

# COREQ Checklist (Tong et al., 2007)

Study Title: Protective Factors for Suicidality: A Qualitative Follow-Up of the Youth and Mental Health Study Cohort

Corresponding Author: Jannike Kaasbøll

Date: 03.05.2025

## Domain 1: Research team and reflexivity

| No. | Item | Guide questions / description | Reported in the manuscript (Section) |
| --- | --- | --- | --- |
| 1 | Interviewer/facilitator | Which author conducted the interview or focus group? | VB (2.2.4) |
| 2 | Credentials | What were the researcher’s credentials? e.g., PhD, MD | Reported in 2.2.5 |
| 3 | Occupation | What was their occupation at the time of the study? | Reported in 2.2.5. |
| 4 | Gender | Was the researcher male or female? | Female (VB) (2.2.5) |
| 5 | Experience and training | What experience or training did the researcher have? | Experience with qualitative analysis and clinical work on suicidality (2.2.5) |
| 6 | Relationship established | Was a relationship established prior to study commencement? | No (2.2.3) |
| 7 | Participant knowledge of the interviewer | What did the participants know about the researcher? | Informed during recruitment (2.2.3) |
| 8 | Interviewer characteristics | What characteristics were reported about the interviewer/facilitator? | Gender, dialect, role in project (2.2.5) |

## Domain 2: Study design

| No. | Item | Guide questions / description | Reported in the manuscript (Section) |
| --- | --- | --- | --- |
| 9 | Methodological orientation and theory | What methodological orientation was stated to underpin the study? | Reflexive Thematic Analysis (2.2.5) Theory (2.1): Bronfenbrenner’s Ecological Systems Theory, Positive Psychology and the Developmental Assets Framework |
| 10 | Sampling | How were participants selected? | Purposive sampling (2.2.3) |
| 11 | Method of approach | How were participants approached? | SMS, followed by phone call (2.2.3) |
| 12 | Sample size | How many participants were in the study? | 14 (2.2.3) |
| 13 | Non-participation | How many people refused to participate or dropped out? Reasons? | 4 (reasons described) (2.2.3) |
| 14 | Setting of data collection | Where was the data collected? | Online (Teams) or by phone (2.2.4) |
| 15 | Presence of non-participants | Was anyone else present besides the participants and researchers? | No (2.2.4) |
| 16 | Description of sample | What are the important characteristics of the sample? | Gender, age, SI/SA history (2.2.3) |
| 17 | Interview guide | Were questions, prompts, guides provided by the authors? Was it pilot tested? | Yes; co-developed with lived experience advisors and pilot tested (2.2.1, 2.2.6) |
| 18 | Repeat interviews | Were repeat interviews carried out? If yes, how many? | No (2.2.4) |
| 19 | Audio/visual recording | Did the research use audio or visual recording to collect the data? | Yes – audio recorded (2.2.4) |
| 20 | Field notes | Were field notes made during and/or after the interview or focus group? | Yes (2.2.5) |
| 21 | Duration | What was the duration of the interviews or focus group? | ~1 hour (2.2.4) |
| 22 | Data saturation | Was data saturation discussed? | Yes (2.2.3) |
| 23 | Transcripts returned | Were transcripts returned to participants for comment and/or correction? | No (2.2.4) |

## Domain 3: Analysis and findings

| No. | Item | Guide questions / description | Reported in the manuscript (Section) |
| --- | --- | --- | --- |
| 24 | Number of data coders | How many data coders coded the data? | One primary coder (VB), with discussions with co-author (JK) (2.2.5) |
| 25 | Description of the coding tree | Did authors provide a description of the coding tree? | Yes – described in analysis procedure (2.2.5) |
| 26 | Derivation of themes | Were themes identified in advance or derived from the data? | Mostly inductive; some deductive elements (2.2.5) |
| 27 | Software | What software, if applicable, was used to manage the data? | NVivo 14, Word, mind maps (2.2.5) |
| 28 | Participant checking | Did participants provide feedback on the findings? | No (not conducted) |
| 29 | Quotations presented | Were participant quotations presented to illustrate the themes/findings? | Yes (2.2.4 and Results section) |
| 30 | Data and findings consistent | Was there consistency between the data presented and the findings? | Yes (Results and 2.2.5) |
| 31 | Clarity of major themes | Were major themes clearly presented in the findings? | Yes |
| 32 | Clarity of minor themes | Is there a description of diverse cases or discussion of minor themes? | Yes – variation and nuances described |
