# Supplementary material for: Protective factors for suicidality: a qualitative follow-up of the youth and mental health study cohort
Source: BMC Public Health. 2025 May 24;25:1920. doi: 10.1186/s12889-025-23131-2 (PMC12102906; doi:10.1186/s12889-025-23131-2)
Supplement: Supplementary file 3 — Supplementary Material 3 [file 12889_2025_23131_MOESM3_ESM.docx]

**Additional file 3**

**Protective factor themes and subthemes with exemplary quotes**

| **Overarching theme** | **Sub-themes** | **Exemplary quotes** |
| --- | --- | --- |
| Meaningful relationships and connectedness | Family support and emotional connection | It was just about having shared it with someone, so she knew (…) Because if at least one person or two wants you in the room, wishes for you to be here and say hi to you and looks after you – then you can get through it (Emma).  It was someone in my family who looked after me and tried to be there for me. I loved her very much too. It might sound weird, but she was someone who always made me cry. She understood that it was difficult to understand what was going on back then, and that it was hard. And that it was ok to be sad. (Nicolas).  I could talk to my friends and my parents, and be open and honest. Not hiding things. I had been hiding things for several years. And that is incredibly hard, like having a rock on your shoulders (Charlotte). |
|  | Close friendships | I am 100% certain that for me it has been crucial to have had good friends around me. Especially my two best friends. They have taken care of me from day one. I don’t think they realize how big a role they have played. (…) For me it has meant everything. It is something I think about daily. I haven’t actually…not yet thanked them for it. I think they know. But…it has meant everything to me. They have included me, came to my home and picked me up. They brought me out to do things. Got my thought on something else. (David).  They accepted me for who I was, and they included me. They backed me up, and in a way…I felt that they made me stronger, in a way (Benjamin).  I think it’s important to find someone you can open up to, because if you do not say anything, nobody will know how you are actually doing. I feel that if you have at least one close friend that you can talk with – who can be open with you too, then you’ll know that you’re not alone, even though you thought you were (Leah). |
|  | Not wanting to hurt loved ones | Mainly I think about how my mother would have reacted. It would just be hurtful. I’ve known many friends who took their lives, and their mothers, they have been completely devastated. It is horrible. (Thomas).  I think I had a lot of awareness of knowing that my family loved me very much. My foster care-family and my sister loved me very much. So I didn’t have the conscience to do it. (Maria).  I had all sorts of thoughts and feelings then, it was… My sister, my little sister, she was pregnant at that time. So I felt that I had a responsibility (Hugo). |
| Support and help from external sources | External helpers | I had conversations with the school nurse, and that helped me a lot. I remember that, she was amazing. (…) There was nothing too strange to talk about with her. Because I remember that it was difficult for me to talk to mum and dad about things, and teachers. But she was kind of like a neutral part. (Alice)  As I was about to jump…and then my acquaintance came running and pulled me down (David).  I probably stood there too long. Suddenly I see someone walking towards me (…) He started talking to me. I think he saved me from doing something stupid that evening. I was certain that my life was going to end there and then. (Leah). |
|  | Seeking professional help | I would have advised myself to seek psychological help much earlier, who could have helped me find the techniques and tools that could have helped back then. And just told me that it is not abnormal to feel this way. It is ok. That would have been the most important thing to convince myself about. And to not have shame attached to it, that made it quite a lot worse (Henrik).  It’s something about rebuilding self-confidence and self-esteem. So I went to a psychologist for a year, in addition to taking anti-depressants for three years (…) There was also a big focus on group therapy. That worked very well. To meet people who are in the same situation as yourself, who have the same thoughts and challenges. You can share experiences and advise. (Charlotte).  It was speaking to the psychologist and getting the third-party view. And it was also good to have a GP during that time, who also was a third-party who listened well and took me seriously. (Benjamin). |
| Changes in the social-environmental context | Engagement and activities and sense of belonging | I had a lot of friends there. And it was kind of an escape from the loneliness I felt here, when I traveled there. We cycled and it was a completely different setting, so the brain also works different. It’s a bit like speaking another language, everything just works differently. I don’t know. Bit smaller environment, but yes. (Thomas).  I think it has helped a great deal. Even now I still play handball. And if I think it has been a difficult or tough day at work, and it’s been a hard week, then I feel that it’s good to be at handball-training. Good to exercise, is what I feel. So I think to have leisure activities, doesn’t have to be sports, but just to have somewhere to go and be in the afternoons (Sofia).  I think, it was the unity in relation to football and that stuff. At least you felt that you had a sanctuary where you mastered something. And at the same time that you had a social community and feeling that you could meet people and hang out in your free time. That you had someone to share a special interest with. And they used a lot of their time and dedicated their time in a similar way to you (Hugo). |
|  | A new start | What happened was, I had to change schools. (…) Things became much better, I got friends. It was a bigger environment, there was more room to be ourselves. (…) I think it was that I felt I had belonging. That I actually belonged somewhere. And that I fitted in. To have mutual interests, and community. (Alice).  It was good to start high school, because none of those who bullied or were mean to you made remarks or anything. They didn’t even go to the same school, so they could be avoided. I think it is important to get away from those who are mean, so you slowly and steadily can rebuild yourself. (Benjamin).  You just felt that you were an empty shell that just did what you had to do at secondary school. But that you started to actually live life when you started high school. I felt more present. I felt that here I fit in, here I can be myself. (Leah). |
| Acceptance and enhanced understanding | Reflective growth through adversity -resilience | I have always thought in hindsight, that what doesn’t kill you makes you makes you stronger. That is at the back of my mind. And that…it is a little victory for oneself to have gotten through it, and…been helped. That simply helps. And to have found the spark in life again (Benjamin).  It's the whole process of not giving up. You must fight. Most of the things you do is difficult. So it’s something about the sense of mastery, to have struggled with something for a long time. That can easily be transferred to other aspects of life. To have spent thousands of hours trying and learning something, and succeeding in the end. So that is a very valuable lesson to have later in life. (Henrik)  It's about turning those thoughts around. To turn around the mindset, when these things come up. It’s been many years since I had those, but…it’s about changing what you think about. It’s not always easy, but…sometimes you just have to decide to prove yourself wrong…and then you must continue to be here. (Oliver). |
|  | Enhanced understanding and societal awareness | I think it is the raised awareness. It has been brought up a lot in the media and… It is very clearly much more accepted to talk about these things today, than it was 20 years ago. Those things have also helped, just hearing people talk about it. Then you understand that you’re not just a black sheep who has that feeling. I think the raised awareness has helped me. (Henrik).  Studying a bit of psychology and things to do with healthcare, you got a bit more understanding of what had happened to yourself. (…) I think when you sit and read, and study those things, you perhaps get a reflection of your own life. That the psyche, it is possible to work on it. (Leah).  You’re in a phrase of life where there’s an awful lot of hormones, there are many feelings. You are uncertain, and others are uncertain too. Life is pretty long, and this phrase you’re in now is a pretty short one. Try to help identifying thoughts about what life can hold afterwords. Everything is much greater. (Nicolas). |
